# Determinants of Fitness App Usage and Moderating Impacts of Education-, Motivation-, and Gamification-Related App Features on Physical Activity Intentions: Cross-sectional Survey Study

**DOI:** 10.2196/26063

**Published:** 2021-07-13

**Authors:** Yanxiang Yang, Joerg Koenigstorfer

**Affiliations:** 1 Chair of Sport and Health Management Technical University of Munich Munich Germany

**Keywords:** smartphone, fitness applications, mHealth, technology acceptance, Unified Theory of Acceptance and Use of Technology 2, physical activity, determinants of app usage, education-related app features, motivation-related app features, gamification-related app features, mobile phone

## Abstract

**Background:**

Smartphone fitness apps are considered promising tools for promoting physical activity and health. However, it is unclear which user-perceived factors and app features encourage users to download apps with the intention of being physically active.

**Objective:**

Building on the second version of the Unified Theory of Acceptance and Use of Technology, this study aims to examine the association of the seven determinants of the second version of the Unified Theory of Acceptance and Use of Technology with the app usage intentions of the individuals and their behavioral intentions of being physically active as well as the moderating effects of different smartphone fitness app features (ie, education, motivation, and gamification related) and individual differences (ie, age, gender, and experience) on these intentions.

**Methods:**

Data from 839 US residents who reported having used at least one smartphone fitness app were collected via a web-based survey. A confirmatory factor analysis was performed, and path modeling was used to test the hypotheses and explore the influence of moderators on structural relationships.

**Results:**

The determinants explain 76% of the variance in the behavioral intention to use fitness apps. Habit (*β*=.42; *P*<.001), performance expectancy (*β*=.36; *P*<.001), facilitating conditions (*β*=.15; *P*<.001), price value (*β*=.13; *P*<.001), and effort expectancy (*β*=.09; *P*=.04) were positively related to behavioral intention to use fitness apps, whereas social influence and hedonic motivation were nonsignificant predictors. Behavioral intentions to use fitness apps were positively related to intentions of being physically active (*β*=.12; *P*<.001; *R^2^*=0.02). Education-related app features moderated the association between performance expectancy and habit and app usage intentions; motivation-related features moderated the association of performance expectancy, facilitating conditions, and habit with usage intentions; and gamification-related features moderated the association between hedonic motivation and usage intentions. Age moderated the association between effort expectancy and usage intentions, and gender moderated the association between performance expectancy and habit and usage intentions. User experience was a nonsignificant moderator. Follow-up tests were used to describe the nature of significant interaction effects.

**Conclusions:**

This study identifies the drivers of the use of fitness apps. Smartphone app features should be designed to increase the likelihood of app usage, and hence physical activity, by supporting users in achieving their goals and facilitating habit formation. Target group–specific preferences for education-, motivation-, and gamification-related app features, as well as age and gender differences, should be considered. Performance expectancy had a high predictive power for intended usage for male (vs female) users who appreciated motivation-related features. Thus, apps targeting these user groups should focus on goal achievement–related features (eg, goal setting and monitoring). Future research could examine the mechanisms of these moderation effects and their long-term influence on physical activity.

## Introduction

### Background

To date, there are 3.8 billion smartphone users worldwide [1], and approximately half of them consider their smartphones as something “they could not live without” [2]. Numerous smartphone apps have been developed to allow users to go beyond basic voice calling and texting to social media, gaming, and managing their health and fitness. In June 2021, 98,406 apps in the Google Play Store and 159,758 apps in the Apple App Store were available to users in the health and fitness category [3,4]. These apps aim to promote physical activity and healthy lifestyles [5,6]. It is important to increase our understanding of the factors that influence users in adopting these apps and subsequent associations with intentions to engage in healthy behaviors—both from the perspective of public health and management (eg, app providers)—because stakeholders in these domains are (or should be) interested in finding ways to promote healthy lifestyles via digitization in general and the use of mobile devices in particular.

The most widely used theoretical frameworks that explain why users adopt or use technology are the technology acceptance model [7] and the Unified Theory of Acceptance and Use of Technology (UTAUT) [8]. The two models focus on the organizational context. In consumer settings, the second version of the UTAUT (ie, UTAUT2) has been developed to explain the acceptance of new technology by individuals [9]. Since the first application of UTAUT2 (studying the acceptance of the mobile internet), it has been used to explain smartphone app adoption and usage [10,11], among other applications. With regard to previous empirical studies on mobile health and fitness apps, important gaps exist in the research. First, previous studies have left out the essential determinants that UTAUT2 incorporates (eg, habit and hedonic motivation). Given the importance of habit [12] and hedonic motivation [13], the sole focus on the four determinants proposed by UTAUT seems insufficient [14,15]. Second, the relationship between the intentions to use fitness apps and to be physically active has not been explored. Assessing the downstream effects of intention to use fitness apps is important, because downloaded but unused apps or apps that are unable to motivate people to become or remain physically active will have fewer health benefits [5,16]. Third, understanding whether different fitness app features moderate the relationships of the UTAUT2 determinants and the behavioral intentions of using the app is lacking. Previous research has categorized app features, such as education-related versus motivation-related features [17], but did not consider their influence on structural relationships that aim to explain app usage intentions and physical activity intentions. Finally, despite the fact that the moderating effects of individual-difference variables (eg, age, gender, and experience) have been theorized and empirically assessed [9], they have largely been neglected in prior research on mobile health and fitness apps [18-21]. However, their relevance was shown in a post hoc meta-analysis, for example, in which age was a significant moderator [22].

This study aims to partially fill these gaps and answer four research questions: (1) What are the relationships between the UTAUT2 determinants and behavioral intentions of individuals to use fitness apps? (2) What is the downstream relationship between the behavioral intentions of using fitness apps and being physically active? (3) Do fitness app features moderate the relationships between the UTAUT2 determinants and the intentions of using fitness apps? (4) Are there individual differences regarding age, gender, and user experience in the relationships between the UTAUT2 determinants and intentions to use fitness apps?

To answer the research questions, we applied and extended the UTAUT2 model in the context of smartphone fitness apps. A sample of 839 individuals was surveyed to test our hypotheses. Path modeling was used to test the hypotheses. In the following, we reviewed the extant literature on determinants of fitness app usage, developed the hypotheses, and presented the methodology of our approach.

### Literature Review

#### Smartphone Fitness Apps

Along with the growing consensus on the health benefits of physical activity [23], a myriad of fitness wearables and smartphone fitness apps have been developed to quantify and promote physical activity. Fitness wearables are “devices that offer training plans, assist with activity tracking, and generally collect and process health-related data” [24], whereas fitness apps refer to “the self-contained programs for smartphones designed for the purpose of getting fit” [25]. This study focused on smartphone fitness apps.

Despite the potential of smartphone fitness apps to deliver cost-effective physical activity and health promotion, their effectiveness has not been sufficiently established [5,16,26,27]. In particular, the effectiveness of fitness apps usage or app-based interventions was modest or short-lived [5,16]. In previous studies, only a limited number of factors considered by researchers have been based on theories or behavior change techniques [16,26,27]. Furthermore, only a small number of fitness apps have undergone rigorous evidence-based evaluations in controlled trials [28]. There are some quality concerns in the reporting of these studies, for example, only a few studies have reported whether fitness apps are based on human behavior change theories [28,29]. Herein, we outline the factors that might predict the behavioral intentions of individuals to use fitness apps (and their downstream effects), building upon theories that have been identified as relevant in the information systems literature, particularly UTAUT2.

#### Determinants of the Behavioral Intentions of Using Fitness Apps

Venkatesh et al [8] developed the UTAUT by integrating eight theories (ie, technology acceptance model, theory of reasoned action, motivational model, theory of planned behavior, combined technology acceptance model, theory of planned behavior model, model of PC utilization, diffusion of innovation theory, and social cognitive theory). According to UTAUT, performance expectancy, effort expectancy, social influence, and facilitating conditions are the four key determinants of behavioral intentions to use technology. In 2012, three additional factors were identified as part of the UTAUT2, namely hedonic motivation, price value, and habit [9]. In the UTAUT2, the individual-difference factors of age, gender, and experience have been identified as important moderators of the relationships between the seven determinants and behavioral intentions. Hew et al [20] applied the UTAUT2 to examine the factors that affect smartphone app adoption in general, considering the moderators of gender and education. They found that all but two factors (ie, social influence and price value) were significant determinants, with habit exerting the strongest influence. Gender and education were nonsignificant moderators. Most important to this research, previous studies used the UTAUT2 to investigate the determinants of behavioral intentions of using fitness-promoting smartwatches [18] and fitness apps [19,30]. However, none of them considered individual-difference factors as moderators, and none of them considered the effect of app features on the proposed relationships.

Specifically, Beh et al [18] found positive relationships among performance expectancy, effort expectancy, facilitating conditions, and hedonic motivation and behavioral intention to use smartwatches for fitness and health monitoring purposes. The authors postulated that perceived vulnerability to developing chronic diseases and perceived severity of chronic diseases would moderate the effects but found only weak support for their hypotheses. Dhiman et al [19] found that effort expectancy, social influence, price value, and habit were positively related to fitness app adoption intentions. They considered self-efficacy to be a predictor of effort expectancy and innovativeness as a predictor of habit; both relationships were significant. Yuan et al [30] did not consider any mediators and found that performance expectancy, hedonic motivation, price value, and habit were predictors of behavioral intentions to continuously use health and fitness apps; however, effort expectancy, social influence, and facilitating conditions were nonsignificant predictors. These studies have important limitations. First, the downstream effects on intentions of being physically active were not assessed in any of the studies. The linkage of fitness app usage intentions and intentions of being physically active is important, because health benefits can only be realized if intended app usage motivates people to become or remain physically active. Second, none of the studies considered app features to be relevant moderators, despite the fact that previous research showed that app features, such as gamification, might moderate the effects of UTAUT2 determinants on app usage intentions [31], and despite the fact that the consideration of risk perception factors (instead of app features) was largely unsuccessful [18]. Third, only one study assessed the moderating roles of age, gender, and experience. However, the authors did not include these variables in the model because of nonsignificant findings [30]. Thus, important similarities with, and differences to the original UTAUT2 studies regarding the influence of age, gender, and experience remain largely unknown. This study aims to fill these gaps partly.

Building upon UTAUT2, we first propose that the seven UTAUT2 determinants relate positively to individuals’ intentions to use fitness apps. Second, we postulate positive downstream relationships with the intention of being physically active. Third, we pose a research question that considers three prominent app features (ie, education, motivation, and gamification related) as moderators of the relationships between the seven UTAUT2 determinants and behavioral intentions of using the app. Finally, we explore the moderating effects of individual differences (ie, age, gender, and experience) on the relationship between the seven UTAUT2 determinants and behavioral intentions to use the app. We have listed the hypotheses in the following sections.

### Hypotheses Development

#### Performance Expectancy

Performance expectancy is defined as the “degree to which using a technology will provide benefits to consumers in performing certain activities” [9]. It was the strongest predictor of behavioral intentions in the original UTAUT study [8] and is a pivotal determinant of new technology acceptance in health care [32,33] and fitness wearables [21,34]. In the context of this study, performance expectancy refers to the degree to which a user believes that using a particular fitness app would help improve their fitness. Previous studies have shown a positive relationship between performance expectancy and intention to use fitness apps [15,30]. As the perception that fitness apps help people reach their fitness-related goals should be of high relevance to users, we propose the following:

Hypothesis 1: performance expectancy is positively related to individuals’ behavioral intentions to use fitness apps.

#### Effort Expectancy

Effort expectancy refers to “the degree of ease associated with consumers’ use of technology” [9], similar to the perceived ease of use as described in the technology acceptance model [7]. In this study, effort expectancy assesses the perceived ease of use of fitness apps. The easier the individuals believe the fitness apps are to use, the higher is their intention to use them. Prior studies have revealed a positive relationship between effort expectancy and behavioral intention to use fitness apps [15,19] and fitness wearables [18,34]. As people should be interested in intuitive and easy app usage, we expect the following:

Hypothesis 2: effort expectancy is positively related to behavioral intentions of individuals to use fitness apps.

#### Social Influence

Social influence is defined as “the extent to which consumers perceive that important others (eg, friends, peers) believe they should use a particular technology” [9]. Social influence plays a particular role when users lack information about their usage [35]. In the context of fitness apps, previous studies have revealed inconsistent results regarding the effect of social influence on behavioral intentions of using fitness apps. It was a positive predictor of usage intentions of students of a Chinese university [15] and Indian users [19], although it did not predict the intentions of college-aged US residents [30]. Given the positive effect of social influence postulated in the original UTAUT2 [9] and the importance of social support in being physically active [36,37], we assume the following:

Hypothesis 3: social influence is positively related to the behavioral intention of individuals to use fitness apps.

#### Facilitating Conditions

Facilitating conditions refer to “consumers’ perceptions of the resources and support available to perform a behavior” [9]. In the context of this research, it reflects the support from resources (eg, ubiquitous internet connection for smartphones) and the required knowledge (eg, experience of smartphone use) to be able to use fitness apps. The original UTAUT2 study [9], as well as studies considering the acceptance of general apps [20] and fitness wearables [18], showed that facilitating conditions increase acceptance. Thus, we postulate the following:

Hypothesis 4: facilitating conditions relate positively to behavioral intentions of individuals to use fitness apps.

#### Price Value

Price value is defined as “consumers’ cognitive trade-off between the perceived benefits of a technology and the monetary cost of using it” [9]. Individuals expect a higher quality of services when they have to pay more for them [30,38]. In the fitness app context, providers offer three main patterns of pricing: free, paid, or freemium (ie, free base app use but additional features need to be paid for). Even if an app can be used for free, individuals might nevertheless consider other cost aspects, such as personal time costs or psychological costs. Previous studies have found a positive relationship between price value considerations and behavioral intentions to use the mobile internet [9], health care wearables [39], and fitness apps [19,30]. Owing to the fact that a high value for a given price can be assumed to be perceived positively by individuals, we propose the following:

Hypothesis 5: price value relates positively to behavioral intentions of individuals to use fitness apps.

#### Hedonic Motivation

Hedonic motivation refers to “the fun or pleasure derived from using a technology” [9]. If the intrinsic motivation of an individual is high, they typically have high levels of hedonic motivation [40]. A meta-analysis revealed that 58% (53/91) of the included UTAUT2-related empirical studies included hedonic motivation as a factor, whereas 81% (43/53) of the studies found a positive relationship between hedonic motivation and behavioral intentions to use the technology [13]. Hedonic motivation has a positive effect on the intention to adopt health care wearables [18,21] and fitness apps [30]. Thus, we suggest that if a user has fun using a fitness app, they are more likely to use it. Hypothesis 6 is as follows:

Hypothesis 6: hedonic motivation is positively related to the behavioral intentions of individuals to use fitness apps.

#### Habit

Habit refers to “the extent to which people tend to perform behavior automatically” and was found to be a positive predictor of behavioral intentions to use the mobile internet [9]. Approximately 35% (23/66) of UTAUT2-related empirical studies utilized habit as a construct [12]. Most importantly, 83% (15/18) of the studies revealed positive associations between habit and intention [12]. In the context of this study, we consider habit to be an important predictor, because smartphones are a central means by which individuals can manage and facilitate their daily lives [2] and because individuals use their smartphone (and potentially fitness apps [19,30]) by habit. We thus propose the following:

Hypothesis 7: habit relates positively with the behavioral intentions of individuals using fitness apps.

#### Downstream Consequence of Behavioral Intentions of Using Fitness Apps

Fitness apps aim to promote user fitness levels. As it is assumable that people who download these apps are (at least partly) committed to reaching this goal, we postulate that higher intentions to use fitness apps relate positively to the willingness of people to be physically active in the future. The claim can be substantiated by consistency theories, arguing that cognitive consistency fosters updates on the expectancy regarding an outcome or a state (here, to be physically active) [41]. However, to date none of the UTAUT2-based studies have examined the relationship between usage intentions of new technology that aims to promote fitness (or health) and the downstream consequence on behavioral intentions to engage in physical activity–related behaviors. Two recent systematic reviews concluded that the effects of fitness apps on physical activity levels are present but are modest in magnitude [5,16]. Previously formed intentions at the individual level might be explanatory variables for these effects. Thus, hypothesis 8 is stated as follows:

Hypothesis 8: behavioral intentions to use fitness apps relate positively to behavioral intentions of being physically active.

#### Moderating Effects of Fitness App Features

Smartphone apps have certain features, that is, the set of operational functions that an app can perform (eg, gaming). The essence of fitness app features may be summarized within behavior change techniques (eg, goal setting, monitoring, and acquisition of knowledge) [42]. In addition, various frameworks of features implemented in fitness apps have been proposed. For example, Mollee et al [43] identified user input, textual or numerical overviews, social sharing, and general instructions as the most implemented features of fitness apps. Rabin and Bock [44] suggested that fitness tracking, tracking of progress toward fitness goals, and the integration of features that increase enjoyment (eg, music) are user-desired features. Other studies focused on the social features of fitness apps (eg, sharing or comparing steps and receiving social support) [45], whereas a review concluded that the evidence of social app features to promote fitness was limited [36].

Conroy et al [17] used an empirical approach to cluster fitness apps in terms of features and used cluster analysis to identify two broad categories, namely, motivation related and education related. Motivation-related app features emphasize the social and self-regulation of fitness (eg, tracking, feedback, social support, goal setting, and reward features). Education-related app features focus on fitness education (eg, instruction, coaching, and learning) [17]. These two clusters do not include gamification-related features, which have become relevant in helping individuals improve their health and fitness [46]. Gamification-related features use game design elements to make the user experience playful and enjoyable [47,48]. In this study, we thus consider gamification-related features besides the motivation- and education-related features of fitness apps.

The literature on apps in general (without a focus on physical activity) has considered app features as moderators of the relationship between acceptance determinants and behavioral intentions of using apps [31,48]. However, it remains unclear whether the UTAUT2 determinants interact with fitness app features to explain the behavioral intentions of using these apps. Such interaction effects might explain the modest effects found in systematic reviews on the effects of fitness apps on physical activity [5,16]. To explore this issue, we formulate the following research question: do fitness app features moderate the relationships between the UTAUT2 determinants and behavioral intentions of using fitness apps?

#### Moderating Effects of Individual Differences

The moderating effects of age, gender, and experience—individual-difference variables—on the relationships between UTAUT2 determinants and behavioral intentions have been proposed and empirically tested in the original UTAUT2 study [9]. In particular, it was theorized that age moderated the relationships between the seven UTAUT2 determinants and behavioral intentions such that the effects are stronger among young (vs old) users for performance expectancy, effort expectancy, and hedonic motivation but weaker for social influence, facilitating conditions, price value, and habit [8,9]. Gender was postulated to moderate the relationship between the seven UTAUT2 determinants and behavioral intentions such that the effects are stronger among women (vs men) for effort expectancy, social influence, facilitating conditions, and price value but weaker for performance expectancy, hedonic motivation, and habit [8,9]. Experience was postulated to moderate the relationships between five UTAUT2 determinants and behavioral intentions such that the effects are stronger among users in the early (vs late) stage of experience for effort expectancy, social influence, facilitating conditions, and hedonic motivation but weaker for habit [8,9]. Three- and four-way interactions of age, gender, and experience were included in the original UTAUT2 study [9]. Despite the fact that the original studies supported these proposed moderator relationships, previous studies on mobile health and fitness apps applying the UTAUT or UTAUT2 did not fully consider them [14,15,18-21,49]. The moderators have been meta-analyzed and suggested as worthy of study [22] or noted as future work [19]. To fill this research gap, we state the following research question: are there individual differences in the relationships between the UTAUT2 determinants and intentions to use fitness apps?

## Methods

### Study Design and Procedure

This study applied a cross-sectional web-based survey design, and the results were reported according to the CHERRIES checklist [50]. Using a convenience sampling technique, we recruited 867 Amazon Mechanical Turk workers in March 2020. This sample size was considered sufficient based on a thumb rule [51], as well as similar studies on fitness app acceptance [19,30]. Participants were limited to healthy adults who were aged between 18 and 65 years, owned a smartphone, and had downloaded at least one smartphone fitness app. Participants were also required to be able to read and understand English and be located in the United States (ie, US residents). Participants who met the eligibility criteria were invited to participate in the Amazon Mechanical Turk online survey, delivered via Qualtrics. All participants were informed about the study procedures via detailed instructions at the beginning of the survey (Multimedia Appendix 1), including the purpose, inclusion criteria, and estimated time needed to complete the survey. After the instructions were provided, informed consent was obtained from each participant. The survey consisted of UTAUT2-related questions, questions that assessed the dependent variables as well as mediators and moderators, and demographics of participants, which were collected at the end of the survey. Each participant was compensated with US $1.50 for their participation. Once 28 incomplete surveys were eliminated, data from 839 respondents were retained for analysis.

This study was conducted in accordance with the ethical standards of the university faculty board, which acts as the local ethics committee for studies outside the Faculty of Medicine, and with the 1964 Helsinki declaration and its later amendments or comparable ethical standards.

### Measures

The UTAUT2 items for the seven determinants and behavioral intentions of using apps were adapted to the context of this study [9]. They were measured on a 7-point rating scale ranging from 1 (strongly disagree) to 7 (strongly agree). The behavioral intentions of being physically active were gauged using two separate measures. First, intentions were measured via an adaptation of the International Physical Activity Questionnaire Short Form [52], which covers a period of 4 weeks in the future. The sum of the values (measured in metabolic equivalent of task [MET] min/week) was calculated according to established data processing guidelines [53]. Second, it was measured using a single question: “To what degree do you want to be physically active in the next four weeks?” (1=not at all; 7=very much) [54]. The individual-difference variables of age and gender were self-reported. Experience was measured with a single item: “When did you download a fitness app for the first time? - () months ago,” as done in the original UTAUT2 study [9].

Participants also rated the features of their most preferred app with importance ratings (1=not important at all; 7=extremely important). Importance ratings were used because apps typically have multiple features and because the features from the perspective of users are important in this study [55]. The items for education- and motivation-related app features were formulated in agreement with previous cluster classifications [17] and substantive content of behavior change techniques [42]. Gamification-related app features were operationalized based on the extant literature on gamification and fitness apps [47,56]. All three app features were measured using three items each. Examples of items are as follows: “How important to you are app features that motivate you to be physically active?” for motivation-related features; “How important to you are app features that educate yourself about how to exercise best?” for education-related features; and “How important to you are app features to enjoy yourself while exercising?” for gamification-related features.

### Statistical Analyses

Normality was evaluated using multivariate skewness and kurtosis [57]. We conducted a confirmatory factor analysis to evaluate the internal reliability, convergent validity, and discriminant validity of the measurement model [58]. For internal reliability, we examined the Cronbach α (>.70) and construct reliability (>0.70). We used the average variance extracted (AVE; AVE>0.50) and factor loadings for convergent validity [59]. Discriminant validity was assessed using the Fornell-Larcker criterion [59] and the heterotrait-monotrait (HTMT) criteria [60]. Various model fit indices were applied, including the normed chi-square statistic (χ^2^/df ratio, value<3.0), Tucker-Lewis index (TLI; TLI>0.90), comparative fit index (CFI; CFI>0.90), root mean square error of approximation (RMSEA; RMSEA<0.05), and standardized root mean square residual (SRMR; SRMR<0.05) [58].

Path modeling (maximum likelihood) was used to test the hypotheses. The variables were mean-centered before the analysis, and gender was coded as a dummy variable (0=female; 1=male). For significant interaction effects between the UTAUT2 determinants and app features, follow-up tests were performed to observe how the moderator changes the hypothesized relationships, as recommended by Dawson [61]. Data analyses were performed using R (RStudio) and the *lavaan* package [62]. The level of significance was set at *P*<.05.

### Participants

A total of 839 participants completed the study. The participants were from 49 US states, with a median of 10 participants per state. They were aged, on average, 37 (SD 10.2) years; 48.3% (405/839) were female; and 51.7% (434/839) were male. Participants were experienced in using fitness apps, as on average they had downloaded the app about 30 months ago. Most participants were White (681/839, 81.2%), employed workers (676/839, 80.6%), married (442/839, 52.7%), and single (322/839, 38.4%). About 66.7% (560/839) reported having a bachelor’s degree or higher, whereas 33.3% (279/839) held an associate’s degree or lower. They were mostly young adults (562/839, 67% aged between 18 and 40 years), and approximately 44.8% (376/839) of them were either overweight or obese. Approximately 76% (638/839) of them had downloaded two or more fitness apps (mean 3.4, SD 2.5). When asked about their preferred fitness app, 14.1% (118/839) stated MyFitnessPal, 13.2% (111/839) stated Fitbit, and 6.2% (52/839) stated Samsung Health (which are among the preferred apps in real-time app rankings under the category of health and fitness in both the Apple App Store and Google Play Store). In total, 159 different apps were mentioned as the preferred apps by the participants. Table 1 shows the sociodemographic characteristics of the participants.

**Table 1:** Sociodemographic characteristics of participants (N=839).

Variables			Values
Age (years), mean (SD)			37.3 (10.2)
Gender (female), n (%)			405 (48.3)
**BMI^a^ (kg/m^2^)**			
	Value, mean (SD)	25.3 (6)	
	Underweight, n (%)	63 (7.5)	
	Normal, n (%)	400 (47.7)	
	Overweight, n (%)	237 (28.3)	
	Obese, n (%)	139 (16.6)	
**Education levels, n (%)**			
	High school degree or below	130 (15.5)	
	Associate degree	149 (17.8)	
	College bachelor’s degree	390 (46.5)	
	Master’s degree	153 (18.2)	
	PhD	17 (2)	
**Marital status, n (%)**			
	Single (never married)	322 (38.4)	
	Married	442 (52.7)	
	Divorced	69 (8.2)	
	Widowed	6 (0.7)	
**Income (US $; gross per year), n (%)**			
	≤15,000	89 (10.6)	
	15,000-24,999	66 (7.9)	
	25,000-34,999	104 (12.4)	
	35,000-49,999	189 (22.5)	
	50,000-64,999	132 (15.7)	
	65,000-79,999	122 (14.5)	
	≥80,000	137 (16.3)	
**Employment, n (%)**			
	Employed	676 (80.6)	
	Self-employed	101 (12)	
	Unemployed	62 (7.4)	
**Ethnicity, n (%)**			
	White	681 (81.1)	
	Black or African American	84 (10)	
	Asian	46 (5.5)	
	Other	28 (3.3)	

^a^BMI was classified according to the US Centers for Disease Control and Prevention’s BMI weight status categories: underweight (below 18.5 kg/m^2^); normal or healthy weight (18.5 to 24.9 kg/m^2^); overweight (25.0 to 29.9 kg/m^2^); and obese (over 30.0 kg/m^2^).

## Results

### Descriptive Statistics and Assumption Tests

Table 2 provides an overview of the descriptive statistics of the variables. The average ratings of the UTAUT2 determinants ranged from 4.26, for social influence, to 6.02, for facilitating conditions. Education-, motivation-, and gamification-related app features were considered important, with the highest ratings for motivation (mean 5.21) compared with gamification- and education-related app features (mean 5 for both). Participant ratings of their behavioral intentions to use fitness apps were above the midpoint of the scale (mean 5.53); intentions of being physically active in the future were very high for both MET values and the ratings on the seven-point rating scale (mean 4589 MET min/week, SD 3137; and mean 6.07, SD 1.05, respectively). All values of skewness and kurtosis were within the suggested criteria (ie, skewness <2 and kurtosis <7 [63]), indicating normality of the univariate distribution.

**Table 2:** Measurement model: descriptive statistics, reliability, and convergent validity.

Constructs^a^ and items			Value, mean (SD)		Skewness^b^		Kurtosis^b^		Reliability						Convergent validity				
									Cronbach α			Composite reliability			Factor loadings			AVE^c^	
**Performance expectancy**										.87			0.87						0.70
	I find the [xx]^d^ app useful in my daily life	5.54 (1.41)		−1.07		0.88								0.84					
	Using the [xx] app helps me accomplish things	5.43 (1.38)		−1.02		0.98								0.86					
	Using the [xx] app increases my physical activity levels	5.50 (1.35)		−1.05		1.08								0.80					
**Effort expectancy**										.89			0.89						0.68
	Learning how to use the [xx] app is easy to me	6.02 (1.11)		−1.41		2.52								0.84					
	My interaction with the [xx] app is clear and understandable	6.01 (1.09)		−1.4		2.62								0.84					
	I find the [xx] app easy to use	6.05 (1.09)		−1.48		2.64								0.86					
	It is easy for me to become skillful at using the [xx] app	5.90 (1.12)		−1.27		2.13								0.77					
**Social influence**										.94			0.94						0.83
	People who are important to me think that I should use the [xx] app	4.30 (1.70)		−0.26		−0.56								0.87					
	People who influence my behavior think that I should use the [xx] app	4.24 (1.73)		−0.25		−0.64								0.92					
	People whose opinions that I value prefer that I use the [xx] app	4.23 (1.72)		−0.29		−0.60								0.94					
**Facilitating conditions**										.77			0.78						0.54
	I have the resources necessary to use the [xx] app	6.08 (1.11)		−1.54		3.03								0.83					
	I have the knowledge necessary to use the [xx] app	6.18 (1.05)		−1.53		2.87								0.83					
	The [xx] app is compatible with other technologies I use	5.80 (1.29)		−1.24		1.61								0.57					
**Hedonic motivation**										.91			0.91						0.78
	Using the [xx] app is fun	5.07 (1.42)		−0.66		0.27								0.93					
	Using the [xx] app is enjoyable	5.24 (1.40)		−0.80		0.50								0.91					
	Using the [xx] app is very entertaining	4.71 (1.58)		−0.48		−0.32								0.82					
**Price value**										.90			0.91						0.76
	The [xx] app is reasonably priced	6.28 (1.13)		−1.7		2.59								0.81					
	The [xx] app is a good value for the money	6.21 (1.14)		−1.5		1.85								0.93					
	At the current price, the [xx] app provides a good value	5.23 (1.15)		−1.72		2.98								0.88					
**Habit**										.80			0.84						0.66
	The use of the [xx] app has become a habit to me	5.34 (1.67)		−1.04		0.33								0.54					
	I am addicted to using the [xx] app	3.65 (1.96)		0.09		−1.25								0.87					
	I must use the [xx] app	3.84 (1.98)		−0.05		−1.24								0.90					
**BI^e^**										.89			0.89						0.73
	I intend to continue using the [xx] app in the future	5.77 (1.37)		−1.41		2.02								0.83					
	I will always try to use the [xx] app in my daily life	5.22 (1.55)		−0.92		0.37								0.85					
	I plan to continue to use the [xx] app frequently	5.61 (1.45)		−1.27		1.46								0.89					
**MO^f^**										.85			0.85						0.65
	How important to you are app features that motivate you to be physically active?	5.13 (1.54)		−0.88		0.31								0.83					
	How important are app features that help you to increase your physical activity levels?	5.38 (1.42)		−1.04		0.88								0.82					
	How important to you are app features that remind you to be physically active?	5.11 (1.63)		−0.87		0.17								0.77					
**ED^g^**										.90			0.90						0.74
	How important to you are app features that educate yourself about how to exercise best?	5.01 (1.62)		−0.77		−0.11								0.86					
	How important to you are app features that tell you how things work when exercising?	4.87 (1.61)		−0.66		−0.30								0.85					
	How important to you are app features that help you do the right things when exercising?	5.11 (1.58)		−0.81		0.12								0.87					
**GA^h^**										.84			0.84						0.63
	How important to you are app features to enjoy yourself while exercising?	5.20 (1.55)		−0.88		0.30								0.86					
	How important to you are app features that gamify the exercise experience?	4.62 (1.83)		−0.51		−0.74								0.68					
	How important to you are app features that make the exercise experience joyful?	5.16 (1.52)		−0.93		0.47								0.88					
**PA^i^**										N/A^j^			N/A						N/A
	Intentions of being physically active during the next 4 weeks (MET^k^ min/week)	4589 (3137)		1.13		1.66								1					
	Intentions of being physically active during the next 4 weeks (1-7 rating scale)	6.07 (1.05)		−1.17		1.68								1					
**EXP^l^**																			
	When did you download a fitness app for the first time? (months ago)	30.07 (25.76)		1.39		2.62		N/A			N/A			1			N/A		

^a^Model fit was satisfactory: χ^2^_564_=2112.2; χ^2^/df=3.8; comparative fit index=0.93; Tucker-Lewis index=0.91; root mean square error of approximation=0.06; and standardized root mean square residual=0.07.

^b^The criteria for skewness (absolute value <2) and kurtosis (absolute value <7) were fulfilled for a sample size greater than 300 (ie, N=839), indicating normality of the univariate distribution [63].

^c^AVE: average variance extracted.

^d^[xx] refers to the brand name of the specified fitness app.

^e^BI: behavioral intentions to use the fitness app.

^f^MO: motivation-related app features.

^g^ED: education-related app features.

^h^GA: gamification-related app features.

^i^PA: Intentions of being physically active. The intentions were measured using the International Physical Activity Questionnaire (metabolic equivalent of task min/week) and a single-item 7-point rating scale. The reported measurement model is based on the first measure.

^j^N/A: not applicable.

^k^MET: metabolic equivalent of task.

^l^EXP: user experience with fitness apps.

### Measurement Model

The overall model fit using MET minutes per week values for physical activity intentions as the dependent variable was found to be satisfactory (χ^2^_564_=2112.2; χ^2^/df=3.8; CFI=0.93; TLI=0.91; RMSEA=0.06; and SRMR=0.07), after excluding one item for facilitating conditions (ie, “I can get help from others when I have difficulties using the [*brand name*] app” with a factor loading of 0.30). The internal reliability, convergent validity, and discriminant validity of the measurement model were evaluated. All Cronbach α and construct reliability values were ≥.77 (ie, above the suggested threshold of 0.70), indicating internal reliability. The AVE and factor loadings were >0.54, in all cases, above the thresholds of 0.50, suggesting convergent validity (Table 2).

Table 3 shows the results of the discriminant validity. First, no cross-loadings were detected among the measurement items. Second, all the square roots of AVE were greater than the relevant interconstruct correlations with two exceptions (ie, performance expectancy: 0.88; and facilitating conditions: 0.87). The HTMT criteria were fulfilled (ie, all HTMT values were ≤0.85) with one exception (performance expectancy: 0.88), but the value is still within the acceptable range between 0.85 and 0.90 [60].

**Table 3:** Discriminant validity of the measurement model: Fornell-Larcker criterion and heterotrait-monotrait ratio.

Variables	BI^a^	PE^b^	EE^c^	SI^d^	FC^e^	HM^f^	PV^g^	HA^h^	MO^i^	ED^j^	GA^k^	PA^l^	Age	GEN^m^	EXP^n^
BI	*.856* ^o^	.879	.646	.414	.623	.604	.473	.795	.423	.218	.241	N/A^p^	N/A	N/A	N/A
PE	.875	*.835*	.651	.464	.594	.694	.405	.747	.635	.368	.378	N/A	N/A	N/A	N/A
EE	.637	.648	*.823*	.181	.785	.435	.614	.341	.321	.147	.179	N/A	N/A	N/A	N/A
SI	.407	.455	.168	*.911*	.135	.536	.057	.616	.366	.366	.375	N/A	N/A	N/A	N/A
FC	.584	.561	.871	.090	*.733*	.394	.678	.281	.278	.146	.178	N/A	N/A	N/A	N/A
HM	.607	.693	.446	.517	.363	*.881*	.254	.650	.515	.458	.571	N/A	N/A	N/A	N/A
PV	.467	.412	.619	.046	.645	.266	*.873*	.181	.199	.077	.097	N/A	N/A	N/A	N/A
HA	.592	.569	.180	.590	.091	.536	.027	*.811*	.470	.316	.366	N/A	N/A	N/A	N/A
MO	.423	.630	.319	.356	.253	.519	.203	.404	*.806*	.683	.712	N/A	N/A	N/A	N/A
ED	.222	.365	.148	.364	.125	.451	.078	.303	.680	*.861*	.632	N/A	N/A	N/A	N/A
GA	.243	.366	.188	.346	.156	.549	.107	.339	.706	.637	*.794*	N/A	N/A	N/A	N/A
PA	.133	.130	.073	.032	.060	.176	.067	.079	.046	.104	.036	*N/A*	N/A	N/A	N/A
Age	.038	.026	.003	−.036	.053	−.034	.084	.001	.055	−.033	−.011	−.035	*N/A*	N/A	N/A
GEN	.019	.064	.118	−.092	.058	−.038	.041	−.016	.157	.063	.096	−.057	.061	*N/A*	N/A
EXP	.095	.043	.159	−.140	.196	.009	.179	−.099	−.040	−.068	−.061	.084	.051	−.011	*N/A*

^a^BI: behavioral intentions to use the fitness app.

^b^PE: performance expectancy.

^c^EE: effort expectancy.

^d^SI: social influence.

^e^FC: facilitating conditions.

^f^HM: hedonic motivation.

^g^PV: price value.

^h^HA: habit.

^i^MO: motivation-related app features.

^j^ED: education-related app features.

^k^GA: gamification-related app features.

^l^PA: intentions of being physically active.

^m^GEN: gender.

^n^EXP: user experience with fitness apps.

^o^Terms in italics along the diagonal are square roots of average variance extracted. Below the diagonal, the lower left metrics test the discriminant validity according to the Fornell-Larcker criterion. Discriminant validity is fulfilled if the square roots of the average variance extracted are larger than the relevant interconstruct correlations. Furthermore, above the diagonal, the upper right metrics refer to the heterotrait-monotrait ratio, where <0.85 or <0.90 indicates good discriminant validity.

^p^N/A: not applicable.

### Structural Model and Hypotheses Testing

**Figure 1:**
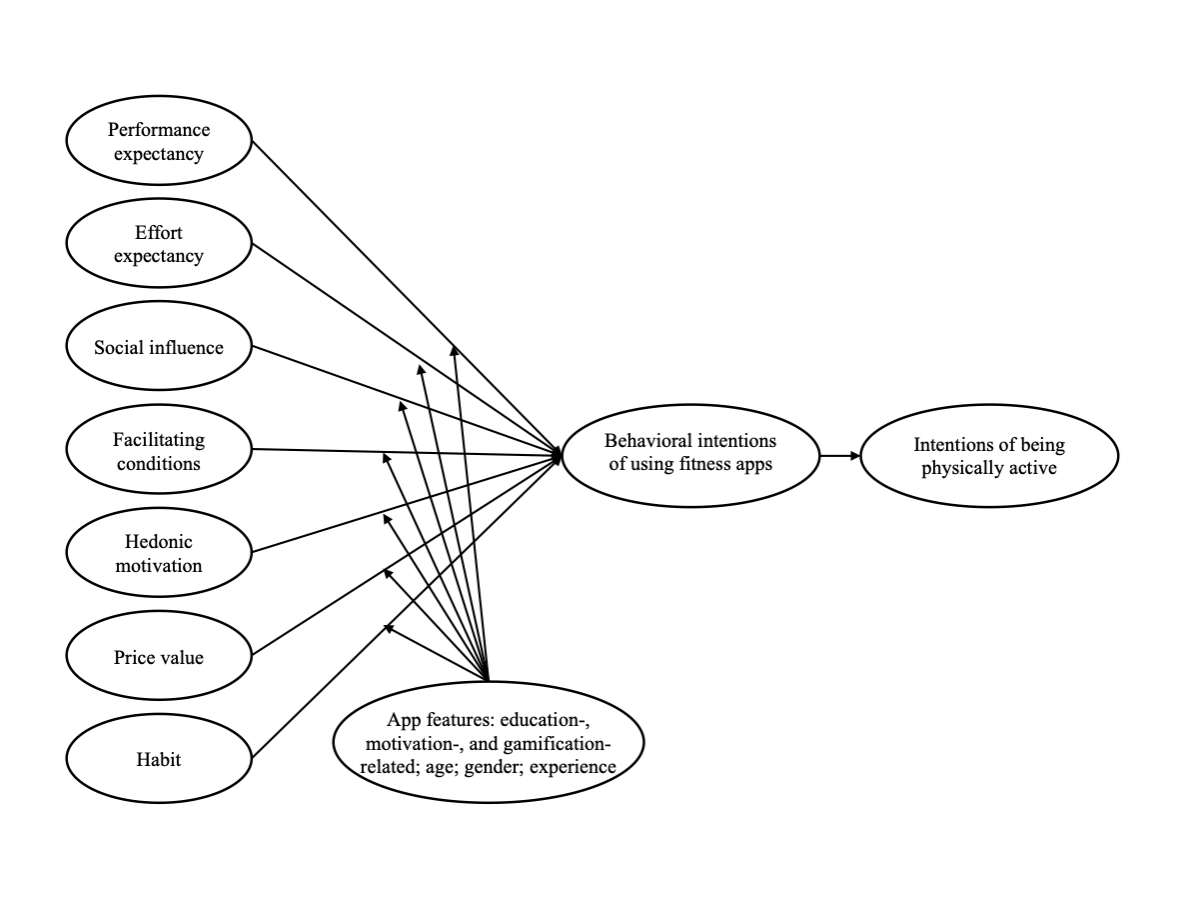
Hypothesized model for predicting behavioral intentions of using fitness apps and engaging in physical activity based on Unified Theory of Acceptance and Use of Technology 2 (UTAUT2) and the consideration of app features. In agreement with the original UTAUT2 study, experience was postulated to not moderate the relationships between performance expectancy and price value and behavioral intentions of using fitness apps.

Path modeling was used to test the hypotheses. The model was established by modeling the hypothesized paths among the UTAUT2 determinants, behavioral intentions of using fitness apps, intentions of being physically active, and the three app features (Figure 1). On the basis of the different measures of intention to be physically active, two models were established. The first model (considering physical activity intentions measured in MET min/week) had an excellent fit (χ^2^_79.00_=97.74; χ^2^/df=1.2; *P=*.08; CFI=0.984; TLI=0.968; RMSEA=0.017; SRMR=0.006). The model fit for the second model (taking into account physical activity intentions measured on a single-item rating scale) was also good (χ^2^_79.00_=179.07; χ^2^/df=2.3; *P*<.001; CFI=0.925; TLI=0.849; RMSEA=0.039; SRMR=0.010). Both models explained 76% of the variance in the behavioral intentions to use fitness apps.

**Figure 2:**
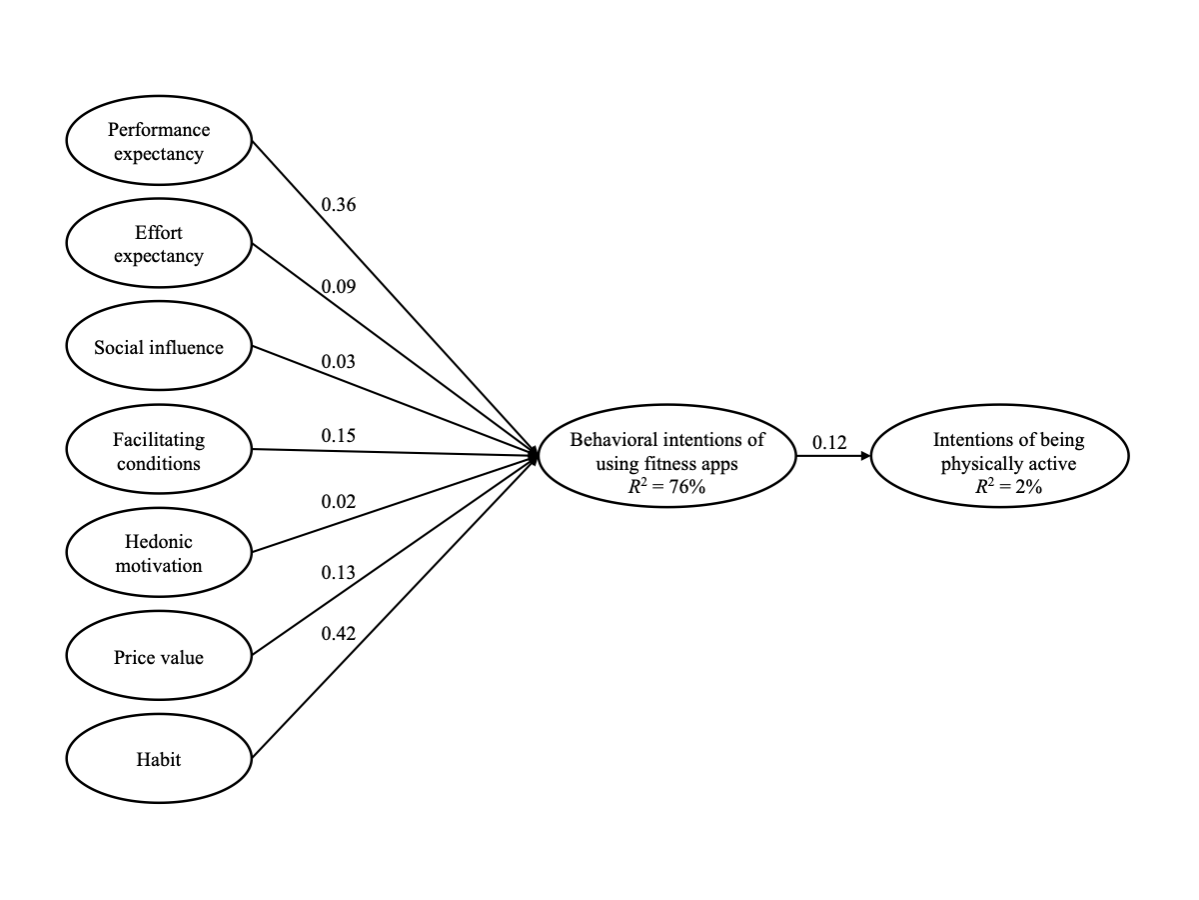
Path modeling results on the relationship between the Unified Theory of Acceptance and Use of Technology 2 determinants and behavioral intentions of using fitness apps, as well as the downstream effects on intentions of being physically active.

In what follows, we first present the results of model 1. Performance expectancy (*β=*.36, *SE* 0.04; *P*<.001), effort expectancy (*β=*.09, *SE* 0.04; *P=*.04), facilitating conditions (*β=*.15, *SE* 0.04; *P*<.001), price value (*β=*.13, *SE* 0.03; *P*<.001), and habit (*β=*.42, *SE* 0.04; *P*<.001) were positively related to behavioral intention to use fitness apps, whereas social influence (*β=*.03, *SE* 0.03; *P=*.37) and hedonic motivation (*β=*.02, *SE* 0.03; *P=*.63) were nonsignificant predictors. Behavioral intentions to use fitness apps relate positively to intentions of being physically active (*β=*.12, *SE* 0.03; *P*<.001), explaining 2% of the variance in physical activity intentions. For model 2, the path coefficients between the UTAUT2 determinants and behavioral intentions of using the fitness app were identical to the results obtained from model 1. Behavioral intentions to use fitness apps relate positively to intentions of being physically active (*β=*.37, *SE* 0.03; *P*<.001), explaining 12% of the variance in physical activity intentions. Thus, hypotheses 1, 2, 4, 6, 7, and 8 were supported, whereas hypotheses 3 and 5 were not supported (Table 4; Figure 2).

**Table 4:** Path coefficients and hypotheses testing for the seven UTAUT2 determinants and app-feature moderators.

Path			*β*^a^ (*SE*)		Z value		*P* value		Hypothesis testing
**UTAUT2** ^b^ **determinants**									
	PE^c^→BI^d^	.36 (0.04)		8.62		<.001		Hypothesis 1 is supported	
	EE^e^→BI	.09 (0.04)		2.02		.04		Hypothesis 2 is supported	
	SI^f^→BI	.03 (0.03)		0.90		.37		Hypothesis 3 is not supported	
	FC^g^→BI	.15 (0.04)		3.55		<.001		Hypothesis 4 is supported	
	HM^h^→BI	.02 (0.03)		0.49		.63		Hypothesis 5 is not supported	
	PV^i^→BI	.13 (0.03)		3.97		<.001		Hypothesis 6 is supported	
	HA^j^→BI	.42 (0.04)		11.52		<.001		Hypothesis 7 is supported	
	BI→PA^k^	.12 (0.03)		3.60		<.001		Hypothesis 8 is supported	
**Education-related features**									
	ED^l^→BI	−.02 (0.03)		−0.89		.37		N/A^m^	
	ED×PE→BI	−.08 (0.03)		−2.46		.01		N/A	
	ED×EE→BI	.01 (0.04)		0.17		.86		N/A	
	ED×FC→BI	.06 (0.04)		1.80		.07		N/A	
	ED×HM→BI	−.02 (0.03)		−0.76		.45		N/A	
	ED×PV→BI	−.04 (0.03)		−1.17		.24		N/A	
	ED×SI→BI	.02 (0.03)		0.70		.48		N/A	
	ED×HA→BI	.08 (0.03)		2.63		.009		N/A	
**Motivation-related features**									
	MO^n^→BI	−.07 (0.03)		−2.34		.02		N/A	
	MO×PE→BI	.10 (0.03)		3.16		.002		N/A	
	MO×EE→BI	.08 (0.04)		2.07		.06		N/A	
	MO×FC→BI	−.11 (0.04)		−2.79		.005		N/A	
	MO×HM→BI	.02 (0.03)		0.69		.49		N/A	
	MO×PV→BI	−.03 (0.04)		−0.72		.47		N/A	
	MO×SI→BI	−.01 (0.03)		−0.47		.64		N/A	
	MO×HA→BI	−.18 (0.03)		−5.46		<.001		N/A	
**Gamification-related feature**									
	GA^o^→BI	−.01 (0.03)		−0.47		.64		N/A	
	GA×PE→BI	−.03 (0.03)		−0.87		.38		N/A	
	GA×EE→BI	−.01 (0.04)		−0.29		.77		N/A	
	GA×FC→BI	−.04 (0.03)		−1.06		.29		N/A	
	GA×HM→BI	.07 (0.03)		2.77		.006		N/A	
	GA×PV→BI	.02 (0.03)		0.68		.49		N/A	
	GA×SI→BI	.01 (0.03)		0.52		.60		N/A	
	GA×HA→BI	−.04 (0.03)		−1.26		.21		N/A	

^a^Unstandardized path coefficient. See Table 5 for the path coefficients of the individual-difference moderators and their interaction effects.

^b^UTAUT2: Unified Theory of Acceptance and Use of Technology 2.

^c^PE: performance expectancy.

^d^BI: behavioral intentions to use the fitness app.

^e^EE: effort expectancy.

^f^SI: social influence.

^g^FC: facilitating conditions.

^h^HM: hedonic motivation.

^i^PV: price value.

^j^HA: habit.

^k^PA: intentions of being physically active, measured in metabolic equivalent of task minutes per week.

^l^ED: education-related app features.

^m^N/A: not applicable.

^n^MO: motivation-related app features.

^o^GA: gamification-related app features.

The testing of the interaction effects of app features and the seven UTAUT2 determinants was performed next (Table 4). Education-related app features moderated the relationships between performance expectancy and behavioral intentions to use fitness apps (*β=*−.08, *SE* 0.03; *P*=.01), as well as between habit and behavioral intentions of using fitness apps (*β=*.08, *SE* 0.03; *P*=.009). Motivation-related app features moderated the relationships between performance expectancy and behavioral intentions of using fitness apps (*β=*.10, *SE* 0.03; *P*=.002), facilitating conditions and behavioral intentions to use fitness apps (*β=−*.11, *SE* 0.04; *P*=.005), and habit and behavioral intentions to use fitness apps (*β=−*.18, *SE* 0.03; *P*<.001). Gamification-related app features moderated the relationship between hedonic motivation and behavioral intention to use fitness apps (*β=*.07, *SE* 0.03; *P=*.006).

The testing of the interaction effects of individual differences and the seven UTAUT2 determinants (Table 5) also revealed that age moderated the relationship between effort expectancy and behavioral intention to use fitness apps (*β*=−.11, *SE* 0.04; *P*=.008). Gender moderated the relationships among performance expectancy and behavioral intention to use fitness apps (*β=*.13, *SE* 0.06; *P*=.03), habit, and behavioral intentions (*β=−*.12, *SE* 0.05; *P*=.02). Experience was a nonsignificant moderator. In addition, the joint moderating tests (three- and four-way effects) taking into account individual differences revealed a significant three-way interaction for age, gender, and hedonic motivation (*β*=−.14, *SE* 0.06; *P*=.02); a significant three-way interaction for age, experience, and effort expectancy (*β*=.09, *SE* 0.03; *P*=.007), and a significant three-way interaction of age, experience, and habit on behavioral intentions to use fitness apps (*β*=−.12, *SE* 0.04; *P*=.004). There were no significant four-way interaction effects.

Subsequently, we conducted follow-up tests to describe how the moderators changed the relationships (Table 6), considering low (−1 SD of the mean) and high (+1 SD of the mean) values of the moderators. First, when education-related features were rated as important, the relationship between performance expectancy and usage intentions was weaker compared with when this feature was rated as unimportant. Second, when education-related features were rated as important, the relationship between habit and usage intentions was stronger compared with when these features were rated as unimportant. Third, when motivation-related features were rated as important, the relationship between performance expectancy and usage intentions was stronger, the relationship between facilitating conditions and usage intentions became nonsignificant, and the relationship between habit and usage intentions was weaker compared with when these features were rated unimportant. Fourth, when gamification-related features were rated as important, the relationship between hedonic motivation and usage intentions was stronger but still nonsignificant compared with when this feature was rated unimportant. Furthermore, the relationship between effort expectancy and usage intentions was positive for younger users but nonsignificant for older users. Finally, the relationship between performance expectancy and usage intentions was stronger among males, whereas the relationship between habit and usage intentions was stronger among females.

**Table 5:** Path coefficients for the individual-difference moderators and their interaction effects.

Path	*β*^a^ (*SE*)	Z value	*P* value
Age→BI^b^	.03 (0.03)	1.26	.21
Age×PE^c^→BI	.03 (0.04)	0.74	.46
Age×EE^d^→BI	−.11 (0.04)	−2.65	.008
Age×SI^e^→BI	−.04 (0.03)	−1.35	.18
Age×FC^f^→BI	.04 (0.04)	1.08	.28
Age×HM^g^→BI	.02 (0.04)	0.45	.65
Age×PV^h^→BI	.01 (0.03)	0.30	.77
Age×HA^i^→BI	.04 (0.04)	1.05	.29
GEN^j^→BI	.06 (0.04)	1.48	.14
GEN×PE→BI	.13 (0.06)	2.20	.03
GEN×EE→BI	.004 (0.06)	−0.07	.94
GEN×SI→BI	−.04 (0.05)	−0.77	.44
GEN×FC→BI	−.06 (0.06)	−1.03	.30
GEN×HM→BI	.06 (0.05)	1.22	.22
GEN×PV→BI	−.05 (0.05)	−1.01	.31
GEN×HA→BI	−.12 (0.05)	−2.34	.02
EXP^k^→BI	.01 (0.03)	0.55	.58
EXP×EE→BI	−.01 (0.04)	−0.38	.70
EXP×SI→BI	−.02 (0.03)	−0.44	.66
EXP×FC→BI	.05 (0.04)	1.15	.25
EXP×HM→BI	.02 (0.03)	0.75	.46
EXP×HA→BI	.01 (0.03)	0.30	.76
Age×GEN→BI	−.02 (0.04)	−0.55	.58
Age×GEN×PE→BI	.10 (0.06)	1.62	.10
Age×GEN×EE→BI	.04 (0.07)	0.63	.53
Age×GEN×SI→BI	.09 (0.04)	1.96	.052
Age×GEN×FC→BI	−.002 (0.06)	−0.04	.97
Age×GEN×HM→BI	−.14 (0.06)	−2.41	.02
Age×GEN×PV→BI	−.02 (0.05)	−0.32	.75
Age×GEN×HA→BI	−.06 (0.05)	−1.16	.25
EXP×GEN→BI	.06 (0.03)	1.99	.047
EXP×GEN×EE→BI	.10 (0.06)	1.72	.09
EXP×GEN×SI→BI	.06 (0.05)	1.32	.19
EXP×GEN×FC→BI	−.04 (0.06)	−0.62	.54
EXP×GEN×HM→BI	−.07 (0.05)	−1.54	.12
EXP×GEN×HA→BI	−.02 (0.05)	−0.53	.60
Age×EXP→BI	.04 (0.04)	1.10	.27
Age×EXP×EE→BI	.09 (0.03)	2.70	.007
Age×EXP×SI→BI	−.02 (0.03)	−0.45	.65
Age×EXP×FC→BI	−.07 (0.04)	−1.71	.09
Age×EXP×HM→BI	.06 (0.04)	1.76	.08
Age×EXP×HA→BI	−.12 (0.04)	−2.85	.004
Age×GEN×EXP→BI	−.002 (0.04)	−0.05	.96
Age×GEN×EXP × EE→BI	−.02 (0.06)	−0.25	.80
Age×GEN×EXP×SI→BI	−.02 (0.05)	−0.41	.70
Age×GEN×EXP×FC→BI	.04 (0.07)	0.58	.56
Age×GEN×EXP×HM→BI	−.09 (0.06)	−1.47	.14
Age×GEN×EXP×HA→BI	.03 (0.05)	0.57	.57

^a^Unstandardized path coefficient. See Table 4 for the path coefficients of the seven UTAUT2 determinants and app-feature moderators.

^b^BI: behavioral intentions to use the fitness app.

^c^PE: performance expectancy.

^d^EE: effort expectancy.

^e^SI: social influence.

^f^FC: facilitating conditions.

^g^HM: hedonic motivation.

^h^PV: price value.

^i^HA: habit.

^j^GEN: gender.

^k^EXP: user experience with fitness apps.

**Table 6:** Slopes for the relationship of the Unified Theory of Acceptance and Use of Technology 2 determinants with behavioral intentions of using fitness apps at different values of the moderator.

Interactions	Low^a^ (−1 SD of mean)			High^b^ (+1 SD of mean)		
	Slope	*t* test	*P* value	Slope	*t* test	*P* value
ED^c^×PE^d^	0.36	8.05	<.001	0.28	2.56	.01
ED×HA^e^	0.42	9.39	<.001	0.50	4.56	<.001
MO^f^×PE	0.36	8.05	<.001	0.46	4.20	<.001
MO×FC^g^	0.14	3.13	.002	0.03	0.27	.78
MO×HA	0.42	9.39	<.001	0.24	2.19	.03
GA^h^×HM^i^	0.02	0.45	.66	0.09	0.82	.41
Age×EE^j^	0.09	2.01	.04	−0.02	−0.18	.86
GEN^k^×PE	0.36	8.05	<.001	0.49	4.47	<.001
GEN×HA	0.42	9.39	<.001	0.30	2.74	.006

^a^Low: low moderators.

^b^High: high moderators.

^c^ED: education-related app features.

^d^PE: performance expectancy.

^e^HA: habit.

^f^MO: motivation-related app features.

^g^FC: facilitating conditions.

^h^GA: gamification-related app features.

^i^HM: hedonic motivation.

^j^EE: effort expectancy.

^k^GEN: gender. The results for females (dummy: 0) are reported as low moderators; the results for males (dummy: 1) are reported as high moderators.

## Discussion

### Principal Findings

The purpose of this study was to examine the influence of the UTAUT2 determinants, as well as the moderating effects of different smartphone fitness app features (ie, education, motivation, and gamification related) and individual differences (ie, age, gender, and experience) on the app usage intentions of individuals and their behavioral intentions of being physically active. The results showed that habit and performance expectancy were the two strongest predictors of intentions of individuals to use fitness apps. The effects of performance expectancy were greater when motivation-related features were rated as important and when education-related features were rated as less important. Moreover, the effects of performance expectancy were greater for males. The effects of habit were greater when education-related features were rated as important and when motivation-related features were rated as less important. Furthermore, the effects of habit were greater for females. Age moderated the relationship between effort expectancy and app usage intention. The intentions of individuals to use fitness apps predicted their intentions of being physically active, using two different means of measuring future physical activity.

### Theoretical Contribution

We contribute to the literature on mobile health and physical activity in several ways. Answering the first research question (*What are the relationships between the UTAUT2 determinants and intentions to use smartphone fitness apps?*), we found positive relationships among habit, performance expectancy, facilitating conditions, price value, effort expectancy, and behavioral intentions to use fitness apps. Habit and performance expectancy were found to be the most important predictors of intention to use fitness apps, consistent with prior studies (eg, habit [19,20,30] and performance expectancy [14,15,30]). Positive relationships have also been identified for effort expectancy [18-20], facilitating conditions [18,20,21], and price value [19,21,30].

Social influence was a nonsignificant predictor of intention [18,20,30]. Interestingly, the latter finding is not due to the high domain-specific experience of users (given the nonsignificant interaction effect of social influence and experience), who might have relied less on peer opinions for their evaluations and intentions than low-experience users. Furthermore, in contrast to the original UTAUT2 study [9] and previous studies [18,20,21,30], but in agreement with Dhiman et al [19], we found a nonsignificant relationship between hedonic motivation and app usage intentions. This may be explained by the high demands of fitness app users on app usage to achieve their physical activity goals, compared with the fun or pleasure derived from the apps. However, focusing solely on the four determinants proposed by the first version of UTAUT [14,15,34] may be insufficient. Habit, in particular, is the strongest determinant linked to the intention to use fitness apps in this study.

Answering the second research question (*What is the downstream relationship between the behavioral intentions of using fitness apps and of being physically active?*), we contribute to UTAUT2-based research by showing that app usage intentions have important downstream consequences. In particular, individuals have greater intentions of being physically active when they have higher intentions to use fitness apps. Assessing the downstream effect of intention to use fitness apps is important, because downloaded but unused apps or apps unable to motivate people to become or remain physically active will have little health effects [5,16]. The positive relationship between fitness app usage intentions and physical activity intentions indicates that app usage might motivate people to become or remain active. The findings thus contribute to previous research into whether, and when, mobile health and fitness apps may help individuals become physically active [64,65]. However, it should be noted that the intentions of individuals to be physically active are affected by numerous correlates and determinants (eg, self-efficacy, sociodemographic variables, sport club membership, among others) [66], and the intention-behavior gap is considerable [67]. Thus, adding these factors and incorporating measurements of actual physical activity may be warranted in the future.

Answering the third research question (*Do fitness apps moderate the relationships between the UTAUT2 determinants and intentions of using fitness apps?*), this study contributes to previous research that categorized app features [17] yet ignored their influence on the structural relationships proposed by the UTAUT2. On the basis of our exploratory analysis, we identified six relevant interaction effects. One of the most intuitive findings was that when motivation-related features were rated as important, the relationship between performance expectancy and intentions was strong. Research into goal achievement [68,69] might explain the interaction effect: individuals who are interested in improving their physical activity levels, or keeping them at certain levels, might use the app exactly for this purpose. Among the three features, motivational elements aim most directly to help users stick to their goals and plans [70]; as there is goal congruence, the effect is strong [71]. When motivation-related features were rated as important, the relationship between facilitating conditions and usage intentions was not significant. This makes sense, because people who lack resources and capacities are more dependent on help from others compared with people who do have these resources and capacities, particularly when motivation features are not considered crucial (ie, motivation might “not be the problem”). In addition, when motivation-related features were important, the relationship between habit and intention was weaker compared with when this feature was unimportant. This finding might indicate that when habits have been formed, features that motivate individuals to be active (eg, reminders) become less important to these app users [72].

This study also found that performance expectancy had a greater effect on usage intentions when education-related features were rated as unimportant. In this case, individuals might be less interested in being educated—an aspect that might distract them from achieving their goals. In addition, the effect of habit on usage intention was stronger when education-related features were rated as important. This may be explained by the fact that habits of individuals are formed best when they are exposed to education-related cues when using an app (eg, how and when to exercise best) [73]. Regarding the interaction between hedonic motivation and gamification-related features, no final conclusions can be drawn. Although research into intrinsic motivation [74] and flow [75] may lead us to propose that intrinsic motivation, as a principal source of enjoyment, may be enhanced by the gamification app features (eg, apps using incommensurate gamification elements [likes]) [76], the follow-up tests did not reach significant levels in this study.

Answering the fourth research question (*Are there individual differences in age, gender, and user experience between the relationships of the UTAUT2 determinants and intentions to use fitness apps?*), we found partly significant, partly nonsignificant moderating effects of age, gender, and experience. First, the relationship between effort expectancy and app usage intentions was stronger among younger individuals, which agrees with the original UTAUT2 study [8,9] and a meta-analysis (ie, age group of those aged 25 to 30 years) [22]. Second, the relationship between performance expectancy and usage intentions was stronger among males, which is consistent with the original UTAUT2 study. In contrast, the relationship between habit and usage intention was stronger among females [9]. Thus, females were not more sensitive to new cues, which might have weakened the effect of habit on behavioral intentions. In the context of fitness apps, females may indeed be prone to cues that help them form health-related habits, because they are interested in health- and body-appearance-related topics. Finally, in this study, experience was a nonsignificant moderator regarding the interaction effects of the UTAUT2 determinants on app usage intentions. Thus, differences in experiences between users might be less relevant today—a time in which smartphone users can easily add and delete new apps and in which users are technology savvy.

### Managerial Implications

This study has implications for smartphone app designers and managers. First, they can be advised to focus on habit formation and performance (eg, goal setting) when designing fitness apps and tailoring them to potential users. Meeting users’ expectations concerning facilitating conditions, price value, and effort expectancy will also increase the likelihood of the app being accepted. Second, practitioners should highlight certain app features that depend on user preferences. For example, motivation-related features are important drivers of app usage intentions for target group users who value performance (education-related features might be less relevant here); habit formation and facilitating conditions are less important to these individuals. Third, health professionals should consider age and gender differences among users with regard to the effects of effort expectancy (age) as well as performance expectancy and habit (gender). Finally, practitioners may also be advised to monitor whether app usage intentions have a positive correlation with intentions of, or even actual, physical activities so that immediate action can be taken when users lose track of their original goals (having already downloaded the app).

### Limitations and Outlook

This study has some limitations. First, the generalizability of our findings is limited. We used a nonrepresentative sample of US residents who owned a smartphone and had previously used fitness apps. Future studies may consider inexperienced people with fitness apps to reveal the influence of UTAUT2 determinants on usage intentions at the early- or preadoption stage. Second, given this research design, we did not consider one specific fitness app, but participants stated their preferred app and rated the features of this app. Thus, we considered a variety of apps (which might be beneficial for external validity, given the myriad of apps on the market [3,4]). Researchers might collaborate with certain providers and use real-world app data and objectively measure actual physical activity to validate our findings. Third, we relied on self-reported physical activity intentions using a single measure and the International Physical Activity Questionnaire Short Form. Overreporting is common for the latter (eg, approximately 84% [77]). Finally, future research could look into the mechanisms of moderation effects on individuals’ behavioral intentions to use apps, incorporate app features into mobile health interventions accordingly, and evaluate their long-term influence on physical activity levels.

## Appendix

Multimedia Appendix 1 Online instructions to participants.

## Abbreviations

AVE: average variance extracted
CFI: comparative fit index
HTMT: heterotrait-monotrait
MET: metabolic equivalent of task
RMSEA: root mean square error of approximation
SRMR: standardized root mean square residual
TLI: Tucker-Lewis index
UTAUT: Unified Theory of Acceptance and Use of Technology
