# Supplementary material for: Determinants of Fitness App Usage and Moderating Impacts of Education-, Motivation-, and Gamification-Related App Features on Physical Activity Intentions: Cross-sectional Survey Study
Source: J Med Internet Res. 2021 Jul 13;23(7):e26063. doi: 10.2196/26063 (PMC8317040; doi:10.2196/26063)
Supplement: Multimedia Appendix 1 [file jmir_v23i7e26063_app1.docx]

**Appendix: Online instructions provided to participants**

Dear participants,

Welcome to this study conducted by the Technical University of Munich. Before you decide whether to take part or not, please take the time to read the following information.

Please only participate in the study if: (1) you own a smartphone and have downloaded an app for physical activity in the past; (2) you are between 18 and 65 years old; (3) you are healthy (namely, without any physical disabilities or chronic diseases that prevent you from being physically active); (4) you are a native English speaker.

The purpose of this study is to explore your habits in relation to smartphone fitness app use and physical activity. Physical activity refers to any bodily movements that result in energy expenditure, which includes not only exercise and sport, but also all activities undertaken while you are working, carrying out house and yard work, traveling, and engaging in recreational activities. Fitness apps for physical activity are typically offered in the Health and Fitness app category in the Google Play Store (Android) or the iOS App Store.

You will be asked to rate/choose different statements according to your feelings, behaviours, and experiences. There are no right or wrong answers. Please try to answer the questions as honest and accurate as possible.

The completion of the study will take about 20-25 minutes, and you will be compensated with $1.50 for your participation. At the end of this study, you will receive a payment onto your account by providing the MTurk payment code provided to you. You must enter this code to receive a payment.

Your participation in this research study is completely voluntary and you may exit at any time. Participation in the study does not involve any risk to you beyond that of everyday life. In addition, all responses are kept confidential and will be analyzed anonymously. Please note that this survey will be best displayed on a laptop or desktop computer. Some features may be less compatible for use on a mobile device.

If you have any questions about this study, or if you are interested in learning about the results of this research study, please contact [name of the researcher] via [email of researcher].
